# Organized Colorectal Cancer Screening Programs in Switzerland – Quo Vadis?

**DOI:** 10.3389/ijph.2025.1608183

**Published:** 2025-04-15

**Authors:** Bianca Albers, Reto Auer, Kevin Selby, Lauren Clack

**Affiliations:** ^1^ Institute for Implementation Science in Healthcare, University of Zurich, Zurich, Switzerland; ^2^ Institute of Primary Healthcare (BIHAM), University of Bern, Bern, Switzerland; ^3^ Center for Primary Care and Public Health (Unisanté), University of Lausanne, Lausanne, Switzerland; ^4^ Division of Infectious Diseases and Hospital Epidemiology, University of Zurich and University Hospital Zurich, Zurich, Switzerland

**Keywords:** colorectal cancer screening, swiss research, primary care, early detection of cancer, implementation research

## Abstract

**Objectives:**

Colorectal cancer (CRC) is among the most commonly diagnosed cancers in Switzerland. Supported by a solid evidence base for CRC screening, cantons have increasingly established organized screening programs. This report summarizes and discusses the state of this program landscape using findings from the Swiss Improving Organized Colorectal Cancer Screening: An Implementation Science Study.

**Methods:**

Semi-structured interviews were conducted with clinical or administrative leads for Swiss CRC screening programs to understand key characteristics, including host organization, enrollment pathways, screening modalities, and program deliverers.

**Results:**

Eleven out of 13 existing or planned programs in 2021 participated, eight of which have been developed since 2020. All programs offer mail invitations to citizens 50–69 years old and fecal immunochemical testing, though positivity thresholds vary. Access to colonoscopy and the role of healthcare providers vary between programs.

**Conclusion:**

Cantonal influences on designing and implementing preventive services allow programs to adapt to local conditions. However, they also challenge opportunities for cross-program learning, efficiencies, and equity. Strengthening the infrastructure connecting programs for shared knowledge building and program improvement will be vital for sustaining Swiss organized CRC screening.

## Introduction

In Switzerland, colorectal cancer (CRC) was the third most commonly diagnosed cancer type in men and second most frequently diagnosed cancer type in women between 2013 and 2017 [1]. During the same period, CRC caused 10% of all cancer deaths in the country and was the third leading cause of cancer mortality across both sexes [1]. While this mortality has decreased in comparison with earlier periods, colorectal cancer remains a considerable burden of disease in Switzerland [2].

The evidence on the effectiveness of interventions for the early detection of CRC is strong. Regular CRC screening, based on the conduct of colonoscopies every 10 years or the biennial use of less invasive stool tests, has been shown to reduce CRC mortality significantly [3–6]. Therefore, health policy developers worldwide have worked to integrate mechanisms into health systems to ensure that more people are screened for CRC regularly. In these efforts, two main approaches can be observed – opportunistic and organized screening [7]. In health systems relying on *opportunistic* screening, CRC screening is encouraged, often through healthcare authorities’ overarching guidelines, recommendations, or funding schemes. However, the responsibility for implementing guideline-adherent CRC screening in these systems is located at the primary care level, where the practices of, e.g., general practitioners (GPs) or community health services, are decisive for whether CRC screening is offered. *Organized* screening builds on explicit policies defining populations to be included within a screening program. These programs are typically organized at a national or regional level and centered on a designated team coordinating program activities, including outreach to eligible populations and program-specific healthcare services. Additionally, organized screening builds on an infrastructure for program quality assurance, e.g., cancer registries [8, 9], adenoma detection rate monitoring, certification of laboratories and healthcare professionals, or timely follow-up on positive fecal immunochemical test (FIT) results [10]. While the superiority of organized over opportunistic screening is not fully established [11–14], strong arguments have been made for organized programs to increase the accessibility of screening to broader populations, making it more likely for more people to be screened and for CRC to be detected early, thereby decreasing CRC mortality [15–18]. This includes organized programs’ potential to reduce health disparities and ensure equitable access to CRC screening through systematic outreach to those who often underutilize screening services, e.g., socioeconomically disadvantaged populations [15, 19].

Following this argument, a growing number of European countries have established organized CRC screening programs. By 2016, 17 European Union member states had established such programs [20], and this number grew to 20 by 2020. The European Commission, in its most recent cancer plan, described ambitions to further enhance access to CRC screening by 2025 [21].

In Switzerland, steps to establish organized CRC screening programs have been taken since 2011 [22], with cantons as the primary driver behind program development. This is due to a health system based on shared responsibilities distributed across federal, cantonal, and municipal levels. While federal entities take a subsidiary role focused on, e.g., health legislation of national importance or the regulation of the Swiss health insurance market, the country’s 26 cantons are the central actors in planning, delivering, and co-financing healthcare services for their residents [23, 24].

For CRC screening, the Federal Office of Public Health (FOPH) ensures that colonoscopies are recognized as an early detection measure and incorporated into the official list of services covered by statutory health insurance plans. The FOPH also ensures FIT are included in the official catalog of analyses to be remunerated, and it approves cantonal CRC screening program-related applications for an exemption from the otherwise mandatory deductibles people pay for health services. However, the FOPH provides no subsidies for program organization, coordination, or operation. These costs remain the sole responsibility of cantons.

Since the 1990s, it has been mandatory for Swiss residents to purchase statutory health insurance from one of more than 50 competing private non-profit insurers that, guided by complex financing mechanisms, operate in the country [20]. During this time, healthcare costs have continually grown [25] making the Swiss healthcare system one of the most expensive [26]. Simultaneously, health insurance plans building on high deductibles as misincentives for forgoing care [27, 28], including preventive services [29, 30], have created doubt about the system’s ability to translate investments into efficient and equitable outputs and outcomes. This makes it pertinent to set up CRC screening as a cost-effective preventive measure [31–33].

Additionally, Switzerland is a culturally diverse country, with four official languages spoken across three regions. French language and culture dominate the Romandy in the West. Northern and Eastern Switzerland are primarily German-speaking, and the area south of the Alps is strongly influenced by Italian/Romansh culture. In breast cancer screening studies, these micro-cultural differences have shown to explain why Swiss-German women’s intentions to engage in mammography screening were lower than those of Swiss-French study participants [34, 35], highlighting that cultural affiliation should be considered in planning and implementing preventive services in Switzerland.

Hence, it is unsurprising that the landscape of CRC screening practices displays cantonal differences. As of March 2025, ten cantons (Appenzell Ausserrhoden & Innerrhoden, Aargau, Glarus, Nidwalden, Obwalden, Schaffhausen, Schwyz, Zug, Zurich), primarily located in the predominantly German-speaking North of the country, had not established a CRC screening program, while the remaining cantons had built (n = 15) or were in the process of preparing one (n = 1) [36]. Most of these programs have emerged since 2019 and are in the early stages of their implementation. Next to federal and cantonal health authorities, this implementation involves additional organizational actors, as the principle of subsidiarity guiding the Swiss health system grants non-governmental and private entities considerable access to the health system [37]. For organized CRC screening programs, this is mirrored in the central involvement of, e.g., *Swiss Cancer Screening* (SCS) and the regional branches of the *Swiss Cancer League* (SCL) in program coordination and delivery. Founded in 2011, SCS is the official Swiss association coordinating all organized cancer screening programs in the country, currently including both colorectal and breast cancer. SCL was formed in 1910 to support people affected by cancer, inform the public about cancer prevention and early detection, and promote cancer research. Health authorities in many cantons have commissioned SCL’s cantonal or regional branches to run one or multiple cantonal CRC screening programs.

Thus, the landscape of these programs in Switzerland has developed substantially in the past decade [36, 38, 39]. While Swiss CRC screening practices and outcomes have been at the center of multiple publications [40–43], only a few organized CRC screening programs have been examined thus far [44–48], which is unsurprising given the young age of most programs.

This study report aims to fill this gap further by providing an overview of the characteristics of existing organized CRC screening programs in Switzerland. It summarizes the first findings from the OCCSI study (Improving Organized Colorectal Cancer Screening: An Implementation Science Study), which focuses on current practices in implementing organized CRC screening programs in Switzerland. Funded by *Swiss Cancer Research*, the study aims to generate cross-cantonal learning about program implementation and support program optimization in Switzerland and other countries with similarly decentralized health policy and practice decision-making structures.

## Methods

### The OCCSI Study

The Zurich cantonal ethics committee confirmed in October 2021 (req-2021-01134) that the OCCSI study does not fall under the Swiss law of research on humans and is exempt from ethical clearance requirements. OCCSI is a *Swiss Cancer Research*-funded study aimed at understanding current practices in implementing organized CRC screening programs in Switzerland. OCCSI builds on a comparative, multiple case study design involving a systematic literature review [49], key stakeholder interviews, document analysis, and an adapted implementation mapping approach [50]. Qualitative data collection was structured into two phases. Phase one focused on generating an overview of the characteristics of all programs. Phase two was an in-depth examination of a purposely selected sample of four programs, the strategies used, and the determinants experienced in program implementation. The findings reported here stem from phase one.

### Program Selection

In this phase, the research team requested contact information for clinical or administrative leads of all existing or planned CRC screening programs (N = 13) in December 2021. Knowing that program teams were, in most cases, small, often not exceeding two to three staff members, the focus on clinical and administrative leads was expected to generate comprehensive insights into program development and characteristics. Programs for which contact information was shared were invited to participate in the study. Following this invitation, non-responding programs received up to two reminders.

### Data Collection and Analysis

Key stakeholder interviews were scheduled for 60 min online using Zoom technology. They involved two interviewers, one of whom led the interview and one taking notes and contributing to the probing of responses. Before each interview, interviewees were sent a program fact sheet (template available in Supplementary Materials). These fact sheets were populated based on program information in publicly available reports or websites, covering program funding, organization and processes, commencement, eligibility criteria, screening modalities, referral pathways, and costs. As part of the interview, these fact sheets were reviewed with interviewees, and information was revised or added as needed, leading to updated fact sheets for all participating programs.

Interviews were transcribed verbatim using a professional transcription agency, and transcripts were shared with interviewees for fact-checking. Based on the information shared during interviews, fact sheets were updated and validated with program representatives in April 2024. The information included in these validated fact sheets was then synthesized descriptively and discussed. The results of this synthesis are presented below.

## Results

### Included Programs

Of the thirteen Swiss CRC screening programs that were existing or in planning at the time of data collection, eleven programs agreed for their information to be shared, one program rejected this sharing, and another program showed to have been put on hold due to the COVID-19 pandemic and internal cantonal processes. Eleven programs were invited to participate in OCCSI; ten agreed, while one remained unresponsive. Later, it showed that one additional program was in planning and was therefore included in the study near the end of data collection. With 10 of the 11 attending programs, interviews were conducted between January and August 2022. Interviews involved nine key program stakeholders, who were clinical (n = 5) or administrative leads (n = 4), with some representing multiple programs. Interviews lasted between 55 and 77 min and were held in German (n = 2), French (n = 2), and English (n = 4). One program preferred to respond to questions in writing. The English version of the questionnaire used for interviews and the program fact sheet were e-mailed to this program, and responses were received 3 weeks later.

### Target Population

All cantonal CRC screening programs in Switzerland are developed for all residents of a canton who are 50–69 years old and at average risk of colon cancer, i.e., do not have a personal or family history of colorectal cancer or specific conditions that represent colon cancer risk factors such as inflammatory bowel disease, or Lynch syndrome. Given the use of opportunistic screening in all cantons, programs emphasize that participants should not have had a colonoscopy in the past ten or a blood-in-stool test in the past 2 years. Table 1 displays the characteristics of these programs.

**TABLE 1:** Characteristics of active Swiss organized colorectal cancer screening programs participating in the “lmproving Organised Colorectal Cancer Screening programmes in Switzerland” study (Switzerland 2021-2023).

Canton	Program commencement	Program host organization	Other screening programs run by host	N eligible program participants	Program enrollment^b^						Primary screening modalities^c^		FIT threshold (ng/mL)	Program deliverers				Program focus
					PRG	GP	GE	PH	GYN	SELF	FIT	COL		GP	GE	PH	GYN	
Basel	2020	Basel Cancer League (KLBB)	BSP^a^	50,000	✓	✓	✓			✓	✓	✓	50	✓	✓			Personalized screening; program reach through FIT
Basel-Land	2023		✗	80,000														
Fribourg	2020	Fribourg Cancer League	BSP	92,600	✓	✓		✓			✓	✓	75	✓	✓	✓		Program reach through FIT
Geneve	2019	Fondation Genevoise pour le Dépistage du Cancer (FGDC)	BSP	123,502	✓	✓	✓	✓			✓	✓	50	✓	✓	✓		Equitable program access
Grisons	2020	Grisons Cancer League	✗	63,000	✓	✓	✓	✓	✓		✓	✓	50	✓	✓	✓	✓	Program reach and coverage
Jura & Neuchâtel	2019	L’Association pour le Dépistage du cancer (ADC) BEJUNE	BSP	70,000	✓	✓		✓		✓	✓		50	✓	✓	✓		Program reach
Lucerne	2022	Canton of Lucerne	✗	114,000	✓	✓	✓	✓	✓	✓	✓	✓	50	✓	✓	✓	✓	
St Gallen	2022	Eastern Switzerland Cancer League	BSP	135,363	✓						✓	✓	50	✓	✓			Program reach through FIT
Ticino	2023	The Ticino Screening Program Center (CPST)	BSP	100,000	✓	✓		✓			✓		50	✓	✓	✓		
Valais	2020	Promotion Santé Valais	BSP	101,000	✓	✓		✓		✓	✓		75	✓	✓	✓		Program reach through FIT
Vaud	2015	Unisanté	BSP	189,000	✓	✓	✓	✓		✓	✓	✓	75	✓	✓	✓		Personalized, risk-based screening; the GP role; shared decision making

^a^ BSP, Breast Screening Program.

^b^ PRG, program; GP, general practitioner; GE, gastroenterologist; PH, pharmacist; GYN, gynecologist; SELF, Self enrollment.

^c^ FIT, fecal immunochemical test; COL, colonoscopy.

### Program Age

With eight programs being developed since 2020, this overview confirms the still relatively young age of Swiss CRC screening programs. This means that most programs have yet to extend invitations to their entire target population, which varies between the most (up to 180,000 individuals) and least populous cantons (50,000 individuals). To obtain this reach, program teams stagger participant invitations such that, e.g., the oldest residents are invited in the first year of program operations to ensure they can benefit from the program before they cross the pre-defined age limit of 69. In contrast, younger age cohorts are gradually invited in the following years until every eligible participant has received at least one invitation.

### Program Host Organizations

Cantonal or regional cancer leagues, formally established as associations and, as such, members of the national SCL, run CRC screening programs in about half of the cantons included in this study (Basel, Basel Land, Fribourg, Grisons, St. Gallen). Associations established for health purposes head the programs in Jura-Neuchâtel and Valais. In contrast, the Geneva program is run by a not-for-profit foundation, Vaud by a university center, and the Lucerne and Ticino programs are an integrated part of cantonal health authorities. For more than half of the included programs, the organizations running the CRC screening program also coordinate an organized breast cancer screening program with staff capacity that is available to the CRC screening program.

### Program Enrolment

Programs have established a broad range of pathways toward program enrollment, ensuring that costs incurred through program participation are billed correctly. The most common is for the program host organization (N = 11), a canton’s GPs (n = 10), or pharmacists (n = 8) to include participants in a CRC screening program. Multiple cantons also allow for online self-inclusion (n = 6) or inclusion through gastroenterologists (GEs) (n = 6) or gynecologists (n = 2). Only one canton, Lucerne, utilizes the entire range of six different enrollment pathways, whereas the average number of pathways used across all cantons is 3.9 (SD = 1.3).

### Screening Modalities

While most cantons allow participants the choice of the FIT every 2 years or a colonoscopy every 10 years in their screening offering, three programs, Jura-Neuchâtel, Ticino, and Valais, have built their screening program on offering the FIT only. However, even for those with a combined service solution, the FIT is often promoted as the “go-to” modality. This is typically due to limited gastroenterology capacity and local preferences for FIT as a genuine low-threshold public health service. This applies to the cantons of, e.g., Fribourg, St. Gallen, and Valais. Cantonal programs also differ in the threshold value chosen to indicate whether a FIT test counts as positive and triggers a follow-up colonoscopy. Fifty ng/mL is the threshold for most cantons, with Fribourg, Valais, and Vaud operating with a slightly higher value of 75 ng/mL.

### Program Deliverers

In all cantonal programs, the healthcare professions delivering program services to participants include GPs and GEs. The role of the GP is to support participants’ decision-making when in doubt about which screening modality to choose, refer patients preferring a screening colonoscopy to a GE, and be involved when positive FIT results require follow-up colonoscopies. Many programs also include pharmacies in their program implementation to ensure low-threshold access to FIT kits. Notably, this involvement can vary in that some cantons (e.g., Lucerne) assign pharmacists a primarily *dispensing* role, while others integrate pharmacies in a broader *consulting* role. Grisons and Lucerne, as the only cantons in Switzerland, have also engaged gynecologists in their screening program to facilitate the referral of women.

### Program Focus

Most programs emphasize *reach* as a critical goal for their current implementation stage and aim to include as many target participants as possible, with some viewing the FIT modality as a key instrument for this endeavor. In this context, the Geneva program described an explicit focus on aiming for *equity* due to the majority (64,4%) of its residents [51] having a migration background. Noteworthy, too, are first attempts made by the Basel and Vaud programs to facilitate personalized screening decisions adjusted to risk and other factors that program participants report, thereby aiming to direct program participants to the least invasive and least costly screening option for them and optimize the use of cantonal gastroenterology capacity.

## Discussion

The findings from this study confirm that the Swiss landscape of organized CRC screening programs has developed substantially in recent years and continues to grow. With a system context of decentralized federalism combined with strong elements of direct democracy, it is unsurprising that programs show similarities and differences. A key factor contributing to differences is the decentralized healthcare system structure, which, in this context, represents a strength as well as a weakness.

On the one hand, Swiss decentralization provides an opportunity for cantons to consider whether an organized CRC screening program is needed at all and to then adapt such programs to local conditions, such as pre-existing cantonal healthcare capacities contributing to a program, including laboratories, gastroenterology clinics, or pharmacies. For example, depending on the gastroenterology capacity available, a program may choose not to offer colonoscopy as a first screening modality, emphasize the FIT as its preferred screening modality, or adjust the threshold value for FIT tests such that the demand for follow-up colonoscopies meets the supply. In this way, decentralization can promote program selection and implementation by offering supportive conditions for optimizing the fit between program characteristics and the local contexts into which it will be embedded.

On the other hand, Swiss decentralization provides cantonal authorities with considerable degrees of power that strengthen the influence of politics on healthcare decision-making as it unfolds between politicians, administrators, and interest organizations [52, 53]. This includes the planning and provision of cantonal preventive services and may contribute to the fact that a cluster of Swiss-German cantons in the North of the country continues to rely on opportunistic CRC screening, leading to their residents being denied a service quality available in neighboring cantons. This raises the normative question of whether cantonal service differences such as these are acceptable to Swiss society, a question that relates to a broader debate about the risks of highly decentralized service systems for equity [52–55], with scholars highlighting that fiscal decentralization may contribute to a decrease in healthcare access and missed opportunities for economies of scale [56].

However, with the continual increase in numbers of organized CRC screening programs that the country has witnessed in the past decade, there is also hope that competitive mechanisms existing between cantons may further alleviate these differences in the coming years. While only two programs existed in 2015, residents in fifteen cantons had access to organized screening by early 2025, with one further program in Thurgau in planning [36]. Using Rogers’ theory of innovation diffusion as a lens [57, 58], observing the experience with organized CRC screening gained by *early adopting* cantons such as Uri [59] and Vaud [38, 46, 60] may have motivated an *early majority* of cantons to implement additional programs in the past decade. The remaining cantons may build on this more comprehensive experience base in the coming time and take steps toward offering organized CRC screening as a *late majority*.

In doing so, it would be relevant to consider how to make existing knowledge and experience with CRC screening program preparation and implementation available to new cantons with limited or no program experience. The decentralized structures of the Swiss healthcare system make it more likely for each canton to develop its CRC screening program from scratch and use considerable resources to explore legislative, administrative, and operational requirements and to design and act on these. Program development efforts undoubtedly also involve cross-cantonal consultations among program peers; however, a pre-designed guideline for establishing new programs could not be identified as part of this study, contributing to a need for local implementation problem-solving capacity independent of external guidance. In host organizations running breast screening programs, the experience gained there was often used to inform CRC screening work. Additionally, *Swiss Cancer Screening,* as a central unit, is tasked with promoting and coordinating the activities of its members. Nevertheless, its operational team of three to four staff is small, necessitating targeted priority setting in providing cantonal support while also conducting policy advocacy at the federal level. This advocacy work includes, e.g., engagement in the CHARTA 2021, a network of professional organizations also including the SCL, Swiss Association of GPs and Pediatricians, Society of Gastroenterology, Pharmacists Association, and Society of Pathology. In working toward broad and easy access for Swiss residents to colon cancer screening through organized programs, the network, for example, applied to the Federal Ministry of Health in 2022 to raise the age for reimbursing colon cancer screening costs to 74 years [61–63]. The final decision is pending. While infrastructures such as these are helpful, their capacity to support cross-program learning to inform and improve ongoing implementation work remains limited. This makes it relevant to consider how to interlink a decentralized system of multiple CRC screening programs to enable mutual learning and continual program optimization.

A growing body of literature points to learning collaboratives (LCs) as a viable strategy for enabling these processes. Rooted in the *Institute for Healthcare Improvement Breakthrough Series* (BTS) model [64], an LC is a multifaceted strategy used to support the implementation of research-supported interventions in routine service settings [65]. An LC brings together constituents from multiple organizations around a shared improvement agenda for a specific intervention. Using site-based data collection and review, expert consultation, leadership engagement, or plan-do-study-act cycles, LC members work together for one to 2 years to optimize local program implementation and build improvement capacity [65]. Learning collaboratives have shown promise as a means to enhance provider as well as patient outcomes in primary [66] and secondary care [67] as well as behavioral [65] and community health [68], especially under supportive contextual conditions [69], and when fidelity to the original BTS model is ensured [65]. In cancer screening settings, LCs have been used to, e.g., re-establish pre-pandemic screening levels after the COVID-19 pandemic among 859 breast, cervical, colon, and lung screening programs in the United States of America (U.S.) participating in a “Return-to-Screening Quality Improvement Collaborative” [70]. Eighty percent of the participating colorectal cancer screening programs reached their screening targets while part of the LC. Increased screening rates (+8%) were also measured in a study of nine U.S. community-based federally qualified healthcare centers participating in an LC focused on enhancing CRC screening rates through local implementation capacity building [71].

In a Swiss CRC screening context, LCs’ focus on supporting (inter-)organizational change [65] could complement an already well-established landscape of provider-centered quality circles [72] in primary care and help strengthen CRC screening practice by building GPs’ capacity for facilitating shared decision-making [73]. Next to the aforementioned topic of *screening participation*, relevant developmental areas suitable as LC learning targets for Swiss programs include, e.g., *shared program implementation standards*, i.e., developing program implementation guidance for future program holders; *equitable program access*, i.e., developing and evaluating strategies for reducing disparities in program participation; *participant engagement*, i.e., finding ways of engaging program participants in program development and evaluation; *gastroenterology capacity*, i.e., creating and testing approaches to building and utilizing this capacity; or *pharmacy involvement*, i.e., enhancing and strengthening the role of pharmacists in program delivery. The learning from focusing on such topics will be valuable for colorectal cancer screening programs and can inform the preparation for additional cancer screening programs, notably lung cancer, which is considered one of the next frontiers in population-based cancer screening [74, 75].

### Strengths and Limitations

This study is unique as it is the first to provide a comprehensive overview of a diverse CRC screening program landscape, capturing variations in structure, scope, and implementation. By synthesizing data across multiple initiatives, it offers new insights into previously unexplored patterns and trends. A further strength of this study is the broad participation of the Swiss organized CRC screening programs that existed at the time of data collection, helping to create a comprehensive overview of common program characteristics.

However, this study reports findings at a single moment in time from a field of cancer screening that is continually developing. For example, in March 2025, when all interviews for this study had been conducted, the Swiss parliament passed an amendment to the Swiss Health Insurance Act by approving a range of measures for cost containment, expanding pharmacies’ opportunities for charging health insurers for their contributions to preventive services, including organized CRC screening programs. The implementation of these changes is scheduled to begin in January 2027 [76]. Furthermore, as highlighted above, when writing this manuscript, an application submitted to federal health authorities for extending the age range of organized CRC screening program participants was pending. Finally, further cantons may have decided to establish a CRC screening program since data collection for this study ended. In using the findings from this study, it is therefore essential to consider the ever-changing context of the Swiss healthcare system.

### Conclusion

The Swiss landscape of organized CRC screening programs has changed extensively in the past decade. While only two programs existed in 2015, residents in fifteen cantons had access to organized screening by early 2025, with one further program in the planning. Existing programs are characterized by several commonalities but also by differences. Most often, these differences represent cantonal efforts to align program characteristics and delivery with local implementation conditions. The Swiss system context of decentralized federalism assigning cantonal decision-makers considerable influence on the design and implementation of preventive services is supportive of these efforts but also challenges opportunities for cross-program learning, economies of scale, and healthcare equity. Strengthening and expanding the infrastructure connecting the different screening programs and supporting shared knowledge building and program improvement will be vital for the sustainability of Swiss organized CRC screening programs and conducive to preparing for the installment of further cancer screening programs in the country.
