# Supplementary material for: Organized Colorectal Cancer Screening Programs in Switzerland – Quo Vadis?
Source: Int J Public Health. 2025 Apr 15;70:1608183. doi: 10.3389/ijph.2025.1608183 (PMC12038373; doi:10.3389/ijph.2025.1608183)
Supplement: Supplementary file 1 [file Table1.DOCX]

| IfIS – Institut für Implementation Science in Health Care IfIS |
| --- |
|  |
|  |

# OCCSI study: CRC screening program information

### Canton:

## Program information – status quo [insert month/year]

| Program website |  |
| --- | --- |
| Program funder(s): Who pays for what? |  |
| Organization(s) running the program |  |
| Does the organization running the program also run other cancer screening programs? | Yes, a breast screening program.  Yes, another screening program than breast.  No |
| Team running the CRC screening program |  |
| Other organizations/ entities involved in running the program |  |
| Program deliverers  *(add comments as needed)* | GPs  Gastroenterologists  Pharmacies  Gynecologists  Laboratory  Pathology  Other |
| Program commencement year |  |
| N inhabitants canton |  |
| Eligibility criteria |  |
| N eligible program participants |  |
| Screening modalities offered | FIT (Fit threshold: [insert value])  Colonoscopy |
| How can eligible participants (request to) receive an invitation? | Program sends invitation letter with reference number unprompted  By phone  Online  Email |
| Who can include individuals in the program once the invitation has been issued? | The cantonal program  GPs  Gastroenterologists  Pharmacists  Gynecologists  Other |
| Does the program stagger program invites in any way? |  |
| How are tests being made available to program participants? |  |
| Participant costs |  |
| General program processes |  |
| Particular program goals or topics that the program pays attention to (other than decrease in incidence and mortality rates) |  |
